# Therapeutic Benefit for Late, but Not Early, Passage Mesenchymal Stem Cells on Pain Behaviour in an Animal Model of Osteoarthritis

**DOI:** 10.1155/2017/2905104

**Published:** 2017-12-24

**Authors:** Victoria Chapman, Hareklea Markides, Devi Rani Sagar, Luting Xu, James J. Burston, Paul Mapp, Alasdair Kay, Robert H. Morris, Oksana Kehoe, Alicia J. El Haj

**Affiliations:** ^1^Arthritis Research UK Pain Centre and School of Life Sciences, University of Nottingham, Nottingham, UK; ^2^Institute for Science and Technology in Medicine, Guy Hilton Research Centre, Keele University, Staffordshire, UK; ^3^Institute for Science and Technology in Medicine, Keele University at RJAH Orthopaedic Hospital, Oswestry, UK; ^4^School of Science and Technology, Nottingham Trent University, Nottingham, UK

## Abstract

**Background:**

Mesenchymal stem cells (MSCs) have a therapeutic potential for the treatment of osteoarthritic (OA) joint pathology and pain. The aims of this study were to determine the influence of a passage number on the effects of MSCs on pain behaviour and cartilage and bone features in a rodent model of OA.

**Methods:**

Rats underwent either medial meniscal transection (MNX) or sham surgery under anaesthesia. Rats received intra-articular injection of either 1.5 × 10^6^ late passage MSCs labelled with 10 *μ*g/ml SiMAG, 1.5 × 10^6^ late passage mesenchymal stem cells, the steroid Kenalog (200 *μ*g/20 *μ*L), 1.5 × 10^6^ early passage MSCs, or serum-free media (SFM). Sham-operated rats received intra-articular injection of SFM. Pain behaviour was quantified until day 42 postmodel induction. Magnetic resonance imaging (MRI) was used to localise the labelled cells within the knee joint.

**Results:**

Late passage MSCs and Kenalog attenuated established pain behaviour in MNX rats, but did not alter MNX-induced joint pathology at the end of the study period. Early passage MSCs exacerbated MNX-induced pain behaviour for up to one week postinjection and did not alter joint pathology.

**Conclusion:**

Our data demonstrate for the first time the role of a passage number in influencing the therapeutic effects of MSCs in a model of OA pain.

## 1. Introduction

Osteoarthritis (OA) is the most common joint disease in adults, and current prevalence is 12% in the population > 60 years, which will escalate over the next 20 years [1, 2]. Although there is controversy in the field, it is acknowledged that a broad spectrum of proinflammatory pathways and catabolic factors contributes to the initiation of OA, which impacts upon both the joint cartilage, synovium, and bone [1]. Pain is one of the first symptoms of knee OA; it can progress to be continuous, reducing movement and quality of life [1].

The mechanisms underlying OA pain involve structural changes and alterations in peripheral transduction and central processing of painful sensory inputs. Current treatments for OA pain have limited efficacy [3], and total joint replacement (TJR) surgery is a common outcome [1]. TJR surgery often reverses central sensitization, indicating that nociceptive output from the joint is fundamental in driving central pain mechanisms [4]. However, surgery is not suitable for patients < 55 years [5], and it remains critical that numbers of people with OA pain reliant on joint replacement as a treatment are reduced.

An alternative approach is the development of more effective cell-based therapies that limit the joint pathology and reduce synovial inflammation, which is significantly associated with OA pain [6].

Mesenchymal stem cells (MSCs) have a potential as a therapy for OA [7–10]. MSCs readily differentiate into bone, cartilage, and adipose cells and release soluble factors (such as growth factors and chemokines) which harbour a regenerative environment through a variety of mechanisms [9, 11]. Animal models mimicking pathological and pain components of OA are widely used [12–14]; intra-articular injection of rat bone marrow-derived MSCs reduced pain behaviour in the absence of an effect on joint pathology in the monosodium iodoacetate model of OA in the rat [15] and had significant chondroprotective and anti-inflammatory effects in a rat surgical model of OA, but pain was not assessed [16]. Similarly, local delivery of adult MSCs was associated with regeneration of meniscal tissue and reduced joint destruction in a caprine model of OA [17]. Increased passage number from 4 to 9 of human adipose-derived adult stem cells increases the chondrogenic potential of cells [18]; whether this translates into improved benefit *in vivo* has yet to be addressed.

Maximising the therapeutic potential of cell-based therapies for the treatment of OA pain requires further understanding of the conditions required to maximise the potential therapeutic effect of MSCs, and knowledge of their sites and mechanisms of action, which requires monitoring of implanted cells within the joint. The aim of the present study was to compare effects of early versus late passage MSCs on pain behaviour, structural changes to the knee joint, and circulating levels of tumor necrosis factor alpha (TNF*α*) and interleukin 10 (IL-10) in a surgical model of OA in the rat. Tracking and imaging of the MSCs within the joint were achieved using magnetic resonance imaging (MRI) of superparamagnetic iron oxide nanoparticles (SPION) internalised by MSCs in a subset of the groups within the study.

## 2. Materials and Methods

### 2.1. Cell Isolation, Expansion, and Characterisation

Early passage bone marrow murine MSCs (P3) were isolated from Balb/c mice, and late passage cells (P9) were isolated from C57Bl/6 mice as previously described [19]. Both sets of cells were fully characterised for membrane receptor expression of CD31, CD44, CD11b, CD45, CD105, and Ly-6A(Sca-1)PE, three lineage differentiation, and colony-forming unit fibroblast assay (CFU-F) (see Supplementary Information).

### 2.2. Magnetic Nanoparticle (MNPs) Labelling

MSCs were labelled with SiMAG (1000 nm; particle size) (Chemicell, Germany). These are commercially available MNPs consisting of a maghemite iron oxide core (Fe_2_O_3_) and an unmodified silica surface with terminal negatively charged silanol groups. Cell were labelled using a passive incubation method as described by Markides et al. [20]. In brief, MNPs were suspended in serum-free CIM (cell isolation media) and added directly to cells in culture. Following a 24-hour incubation period, cells were washed three times with phosphate buffered saline (PBS) to remove noninternalised MNPs.

### 2.3. CellTracker™ CM-DiI Fluorescent Dye Labelling

A 1 mg/ml stock solution of the red fluorescent CellTracker CM-DiI (Molecular Probes, UK) was prepared in dimethyl sulfoxide (DMSO). MSCs were trypsinized, washed with PBS, and incubated with CM-DiI (2.5 *μ*l of stock per 1 ml of PBS) for 5 minutes at 37°C, and then for an additional 15 minutes at 4°C, in darkness.

Unincorporated dye was then removed by centrifugation at 300*g* for 5 minutes and 2 washes in PBS. Cells were resuspended in serum-free IMDM (Iscove's Modified Dulbecco's Medium) and maintained at 4° until injection.

### 2.4. MSC-Conditioned Medium Studies

Cells used for conditioned medium studies were previously isolated from C57Bl/6 and Balb/c mice and expanded *in vitro*. For testing, conditioned medium was prepared using murine bone marrow-derived MSC at P3 and P10. Conditioned medium was prepared using serum-free IMDM (SF-IMDM) (GIBCO, Life Technologies) with no supplements added as described previously [21]. Briefly, cells were expanded to confluence using cell expansion medium (CEM, comprising IMDM with 9% FBS, 9% Horse Serum, and 1% Pen/Strep). Flasks were then rinsed three times with DPBS and once with SF-IMDM before adding 12 ml SF-IMDM. Following this, flasks were incubated at 37°C, 5% CO_2_ with SF-IMDM without cells used for controls. After 48-hour incubation, the medium was removed and centrifuged for 5 minutes at 1200*g* to remove cell debris. 11 ml supernatant was then passed through 3 kDa centrifugal filters (amicon ultra 15 centrifugal filter tubes, Merck Millipore, Hertfordshire, UK) at 4000*g* for 40 minutes at 4°C, and residual supernatant was removed from filters and immediately frozen to −80°C until testing.

### 2.5. Rat Model of Osteoarthritis Pain

Male Sprague Dawley rats (weighing 160–190 g) were purchased from Charles River UK. Studies were carried out in accordance with UK Home Office Animals (Scientific Procedures) Act (1986) and the guidelines of the International Association for the Study of Pain. Further, all works were conducted under Home Office project licence number 40-3647. Studies were undertaken in a blinded fashion. Rats underwent medial meniscal transection (MNX), or sham surgery, as previously described [22]. The surgical MNX model of OA in rats has been demonstrated to induce symptoms comparable to that seen in human OA for pain behaviour, weight-bearing asymmetry, and disease pathology, through synovitis, pathology of the subchondral bone, and chondropathology, as well as the development of osteophytes [13, 22–24]. Rats were anaesthetised with isoflurane (3% induction, 2–2.5% maintenance; 1 L/min O_2_), and local anaesthetic EMLA cream was applied to the left hind limb. A full thickness cut through the medial meniscus of the left knee was performed. Sham-operated rats had their meniscus exposed, but not transected. Recovery from anaesthesia was monitored, and weight gain and general behaviour were monitored throughout the postinjury period.

### 2.6. Intervention Studies and Pain Behaviour

Baseline measurements were taken prior to surgery (day 0) and from day 3 onwards. Behavioural assessment of changes in weight distribution and sensitivity to mechanical stimuli applied to the hindpaw were performed for up to 42 days postsurgery (see Supplementary Information). Two separate intervention studies were undertaken at 14 days postsurgery (MNX and sham). Prior to treatment, rats were stratified according to weight bearing and paw withdrawal thresholds (PWTs) (days 3–14) to ensure balanced groups. Under brief isoflurane anaesthesia (3% 1 L/min O_2_), rats received one intra-articular injection.

#### 2.6.1. Study 1: Late Passage MSCs (P.10)

MNX rats received intra-articular injection of either 1.5 × 10^6^ mesenchymal stem cells labelled with 10 *μ*g/ml SiMAG (MSC-MNP; *n* = 11 rats), 1.5 × 10^6^ mesenchymal stem cells (MSC-VEH; *n* = 12 rats), or serum-free media (SFM; *n* = 12 rats). Sham-operated rats received intra-articular injection of serum-free media (SFM; *n* = 8 rats).

#### 2.6.2. Study 2: Early Passage MSCs (P.3) versus Steroid Treatment

MNX rats received intra-articular injection of 200 *μ*g/20 *μ*L Kenalog (*n* = 8), 1.5 × 10^6^ MSC (*n* = 10 rats), or serum-free media (*n* = 10 rats). Sham-operated rats received serum-free media (SFM; *n* = 8 rats).

Following intra-articular injection, rats recovered from anaesthesia and were returned to the home cage. Weight bearing and PWTs were assessed on days 21, 28, 31, 35, and 38 post sham/MNX surgery. Experiments were terminated on day 42.

### 2.7. Magnetic Resonance Imaging (MRI)

The *in vivo* MRI visibility threshold was determined previously by intra-articular injection of either 1 × 10^6^ or 2 × 10^6^ MSCs labeled with 0, 1, 5, and 10 *μ*g/ml SiMAG into the joint of nonarthritic cadaveric 18-week-old Wistar rats. Rats were MR imaged using a Brucker 2.3 T animal scanner with the following sequence parameters; T2-weighted GEFI sequences, TR = 700 ms, TE = 5.5 ms, Flip angle = 30° and FoV = 7.9 × 7.9 cm, and matrix size = 256 × 192 to determine the location of the MNPs. MR images and signal loss profiles were compared to the untreated control groups and also between the treatment groups. Study 1 rats were sacrificed on day 42 and immediately MR imaged using the same system described above. Signal loss profiles were obtained and compared across all the groups. For details on data analysis, see Supplementary Information.

### 2.8. Histology

At sacrifice, tibiofemoral joints were removed and postfixed in neutral buffered formalin (4% formaldehyde) decalcified in ethylenediaminetetraacetic acid (EDTA) [24]. Histomorphometry was performed by an observer blinded to treatment. Coronal tissue sections (Osteoarthritis Research Society International (OARSI) guideline for histological assessment for OA in the rat) were cut at 5 *μ*m [25].

Haematoxylin and eosin (H&E) stained sections were scored for joint morphology [26]. To validate the MRI results from study 1, midsagittal serial sections (4 *μ*m) were obtained and stained with H&E and the fluorescent dye DAPI (1 : 200 dilution prepared in PBS) in order to visualise implanted CM-DiI-labelled MSCs (see Supplementary Information).

### 2.9. ELISA

At sacrifice, blood was taken via cardiac puncture, aliquots spun for 20 minutes at 1000*g*, and serum supernatant was collected. Serum samples were diluted 2-fold, TNF*α* using 75 *μ*l with calibrator diluent (R&D Systems, RD5-17), IL-10 using 50 *μ*l with assay diluent (R&D Systems, RD1-21), and *β*NGF using assay diluent made up using 10% heat inactivated FBS (Life Technologies Ltd., 10500-064). Serum levels of TNF*α* and IL- 10 were determined using commercially available Enzyme-linked immunosorbant assay (ELISA) kits (RnD Systems, Minneapolis MN) as per manufacturer's instructions. Each serum sample was repeat tested *n* = 2, and absorption read at 450 nm with correction at 540 nm applied. Proprietary kits for measurement of *β*NGF in rat serum were not available, so components were sourced individually with the basic application using Duoset ELISA Development Kit (R&D Systems Europe, Ltd., DY556) and recommended components as per kit instructions (all R&D Systems Europe Ltd.) except for DPBS (GIBCO, Life Technologies, 14190-169).

### 2.10. Statistics

Data were analysed using GraphPad Prism 5.0. All data were tested for normality and for nonparametric testing; Kruskall-Wallis one way ANOVA with Dunn's post hoc testing or 2-way ANOVA with Tukey's post hoc testing was applied where appropriate, with probability values considered significant at ^∗^*p* < 0.05, ^∗∗^*p* < 0.01, and ^∗∗∗^*p* < 0.001.

## 3. Results and Discussion Results

### 3.1. MSC Selection and Characterisation

Murine MSCs were freshly isolated and expanded *in vitro* to passage 3 (early passage) or passage 10 (late passage) with passaging taking place at 80–90% confluence. Stem cell properties were confirmed with flow cytometry for a panel of recognised MSC markers (CD105^+^, Sca-1^+^, CD31^−^, CD11b^−^, CD45^−^, CD44^+^, and CD34^−^) (Supplementary Information Figure 1) and selected for use. Cells were successfully differentiated towards adipogenic, osteogenic, and chondrogenic lineages after 21 days in culture with relevant differentiation media (Supplementary Information Figure 2). CFU-F assay was used to assess the proliferative capacity of the cells being expanded in culture, and the results showed that late passage cells retained a high proliferation rate in culture (early passage 49 ± 18% versus late passage 51.5 ± 12%). Early and late passage cells were cultured for conditioned medium collection, and levels of key cytokines (IL-10, TNF*α*, and *β*NGF) were measured (Supplementary Information Figure 3). Levels of IL-10 in the conditioned medium were minimal and did not vary between early and late passage cells. Levels of *β*NGF were significantly higher in conditioned medium from late passage MSC, compared to early passage MSC (Student's *t*-test, *p* < 0.05, *n* = 6). TNF*α* was not detected in the conditioned medium under either condition (Supplementary Information Figure 3).

### 3.2. MRI of MSCs Labelled with SiMAG *In Vivo*

The presence of iron oxide MNPs was detected as a decrease in signal intensity when MR imaged using T2-weighted MRI sequences. This signal loss is portrayed visually as black areas on grey scale MR images, referred to as hypointense regions. The optimal cell number and SiMAG ratio to ensure good MRI visibility over a prolonged period of time were determined to be 1.5 × 10^6^ cells labelled with 10 *μ*g/ml of SiMAG (Supplementary Information Figure 4). This labelling combination allows for improved visibility thresholds over 1 × 10^6^ whilst minimising excessive blooming as seen with 2 × 10^6^ cells and was taken forward for subsequent *in vivo* MRI tracking studies.

### 3.3. Effects of MSC Treatment versus Steroid on Pain Behaviour in a Model of OA

**Figure 1:**
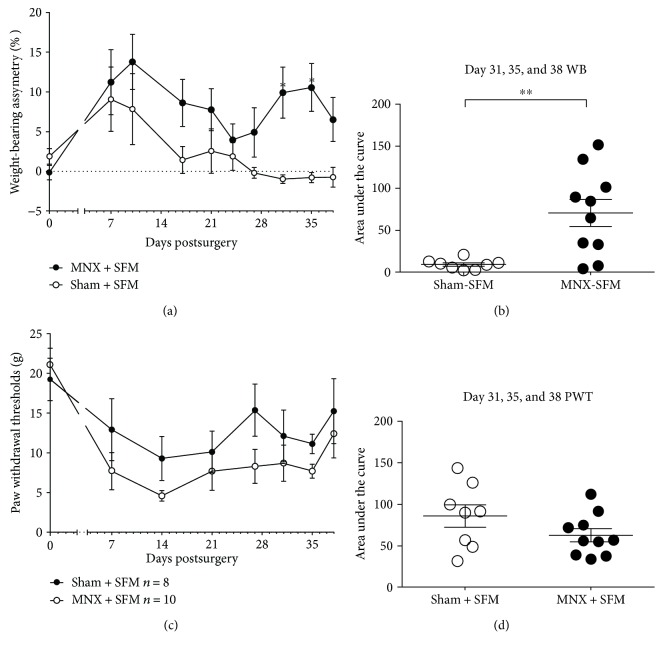
MNX surgery for induction of OA pain, or sham surgery, was performed on day 0. (a) MNX rats receiving SFM exhibited significant differences in weight-bearing asymmetry, compared to the sham-SFM rats. (b) Area under the curve (AUC) data calculated for days 31, 35, and 38 postsurgery revealed significant weight-bearing asymmetry in MNX rats at the later timepoints of the model. (c) Paw withdrawal thresholds were not significantly different in MNX rats, compared to sham controls at individual timepoints, or following AUC analysis. (d) Statistical comparison of the groups at each timepoint: two-way ANOVA with Bonferroni's post hoc. Comparison of AUC used a Mann–Whitney's nonparametric unpaired *t*-test. ^∗^*p* < 0.05, ^∗∗^*p* < 0.01 MNX versus sham. Data are mean ± SEM, *n* = 8–10 per group.

Both sham surgery and MNX surgery resulted in early changes in weight bearing on the operated hindlimb. By day 14 postsurgery, there was a clear difference between the extent of weight-bearing asymmetry between the sham and MNX groups, indicative of pain behaviour in the MNX group. At days 28–38, weight-bearing asymmetry remained significantly increased in the MNX group, but had returned to baseline in the sham group (Figure 1(a)). Area under the curve analysis of the last three timepoints tested (days 31–38) revealed a significantly greater weight-bearing asymmetry in the MNX group compared to the sham control group. Consistent with the studies in our group, hindpaw withdrawal thresholds (PWTs) were lowered in both sham and MNX rats following surgery (Figure 1(c)). There were no significant differences in the PWTs between the two groups of rats (Figure 1(d)).

**Figure 2:**
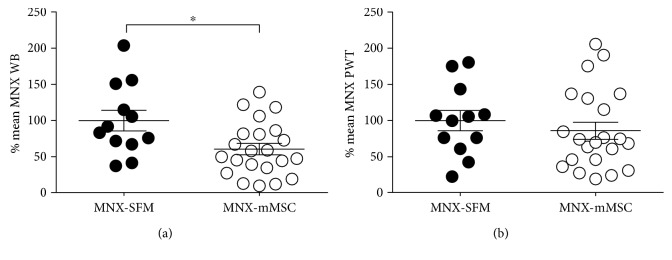
Rats received an intra-articular injection of 1.5 × 10^6^ late passage mouse mesenchymal stem cells (mMSCs) from the bone marrow of Balb/c mice in 50 *μ*L serum-free medium (SFM) or 50 *μ*L SFM (vehicle) on day 14 post MNX surgery. (a) Intra-articular injection of MSCs significantly altered MNX-induced changes in the weight-bearing asymmetry. (b) Paw withdrawal thresholds in MNX rats were unaltered by the treatment. Data are expressed as a % of the mean MNX-SFM AUC for three timepoints (31, 35, and 38 days) postsurgery. Statistical analysis used a Mann–Whitney nonparametric unpaired *t*-test. Data are mean ± SEM, MNX-SFM (*n* = 11), MNX-MSC (*n* = 22). WB: weight-bearing asymmetry; PWT: paw withdrawal thresholds. ^∗^*p* < 0.05 MNX-MSC versus MNX-SFM.

**Figure 3:**
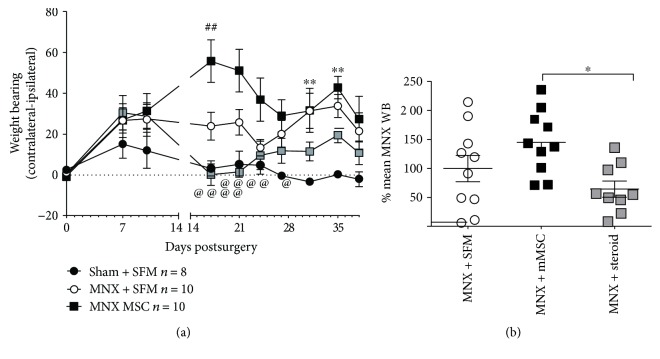
(a) Timecourse of the effects of intra-articular injection of the steroid Kenalog (200 *μ*g/20 *μ*L) versus 1.5 × 10^6^ of early passage mMSC on weight-bearing asymmetry. Rats received the active treatments or 50 *μ*L SFM (vehicle) on day 14 postsurgery. Kenalog had a rapid inhibitory effect on MNX-induced weight-bearing asymmetry at 17 and 21 days post model induction, compared to the MNX-SFM group. mMSC treatment significantly increased weight-bearing asymmetry at early timepoints (days 17 and 21) post model induction. At later timepoints, weight-bearing asymmetry was comparable between the MNX-MSC group and the MNX-SFM group. Data was analysed using a 2-way ANOVA with Tukey's post hoc test. ^∗∗^*p* < 0.01 MNX-SFM versus sham-SFM, ##*p* < 0.01 MNX-MSC versus MNX-SFM; @*p* < 0.05, @@@@*p* < 0.0001 MNX steroid versus MNX-MSC. (b) Area under the curve (AUC) analysis of the effects of intra-articular injection of steroid Kenalog versus 1.5 × 10^6^ of early passage mMSC on MNX-induced weight-bearing asymmetry for the last three timepoints (days 31, 35, and 38). mMSC treatment did not alter weight-bearing asymmetry in MNX rats. Although there was a trend towards an inhibition of weight-bearing asymmetry by Kenalog, this was only significantly compared to the MNX-mMSC group. Data are expressed as a % of the mean MNX-SFM AUC for timepoints 31, 35, and 38 days postsurgery. Statistical analysis used a Kruskal-Wallis test with Dunn's post hoc, ^∗^*p* < 0.05. Data are mean ± SEM, *n* = 9-10 per group.

The effect of intra-articular injection of 1.5 × 10^6^ of late passage MSCs on established pain behaviour in the MNX model was determined. MSC treatment did not alter weight-bearing asymmetry in the week (days 14–21) immediately following treatment (data not shown). By contrast, there was a significant reduction in weight-bearing asymmetry at later timepoints (days 31, 35, and 38) in the MNX group treated with late passage MSCs (Figure 2(a)). PWTs were not altered by the intra-articular injection of late passage MSCs (Figure 2(b)). The effects of intra-articular injection of early passage MSCs versus a steroid treatment on pain behaviour in the MNX model were determined in separate groups of rats. Intra-articular injection of early passage MSC at day 14 resulted in a robust and significant increase in weight-bearing asymmetry at days 17 and 21 post model induction (Figure 3(a)). At later timepoints (days 29–38), weight-bearing asymmetry was comparable between the MNX-MSC group and the MNX-SFM group (Figure 3). Intra-articular injection of the steroid Kenalog at day 14 in MNX rats resulted in a complete reversal of weight-bearing asymmetry at days 17 and 21 (Figure 3). At later timepoints (days 29–38), Kenalog ceased to inhibit weight-bearing asymmetry and there were no differences between the MNX-SFM control group and the MNX Kenalog treatment group (Figure 3(b)).

### 3.4. Joint Pathology, Inflammation, and Pain-Related Cytokines

**Figure 4:**
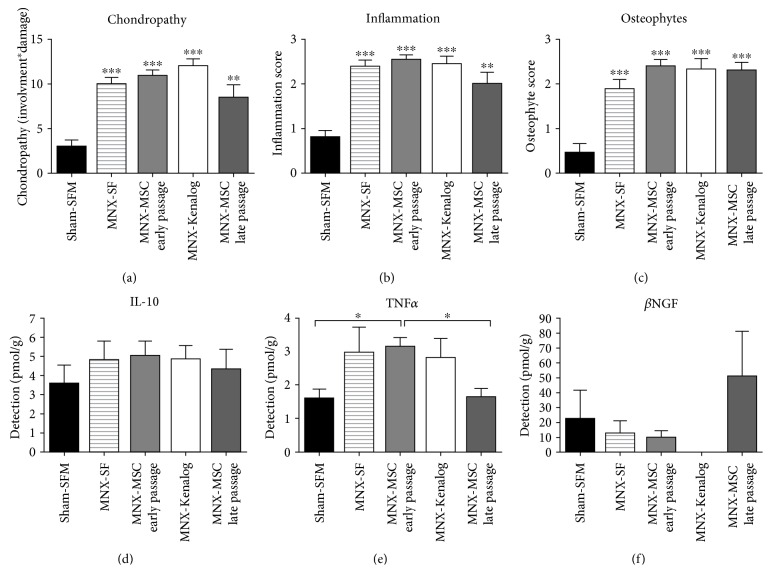
(a–c) MNX surgery was associated with significant chondropathy (a), joint inflammation (b), and increased presence of osteophytes (c), compared to sham controls at 42 days postsurgery. None of the treatments in MNX rats significantly altered the extent of chondropathy, inflammation, or osteophyte number. Statistical analysis used Kruskal-Wallis test with Dunn's post hoc, ^∗∗^*p* < 0.01, ^∗∗∗^*p* < 0.001. Data are mean ± SEM, 2–4 sections per rats were analysed, and total numbers of sections are sham-SFM: 37; MNX-SFM: 55; MNX-MSC early passage: 44; MNX Kenalog: 42; MNX-MSC late passage: 20. (d–f) Serum levels of cytokines in MNX- and sham-operated rats at 42 days postsurgery. There were no differences in IL-10 expression between the treatment groups (d). There was a significant increase in serum TNF*α* in the MNX-MSC early passage treatment group, compared to the sham-SFM controls and MNX-MSC late passage treatment group (e). There were no significant differences in serum *β*NGF expression between the groups. Statistical analysis used Kruskal-Wallis test with Dunn's post hoc, ^∗^*p* < 0.05, ^∗∗∗^*p* < 0.001. Data are overall mean ± SEM, whilst mean values from duplicate samples per rat were analysed; total numbers of mean values are sham-SFM: 15; MNX-SFM: 22; MNX-MSC early passage: 10; MNX Kenalog: 9; and MNX-MSC late passage: 23.

Analysis of joint histology at the end of the study revealed that MNX surgery resulted in a significant chondropathy score (Figure 4(a)), inflammation score of the synovium (Figure 4(b)), and an increase in the number of osteophytes (Figure 4(c)). At this final timepoint, none of the treatments significantly altered joint chondropathy or inflammation (Figures 4(a), 4(b), and 4(c)). Serum levels of three cytokines were measured in the different treatment groups at the end of the study (day 42 postsurgery). There were no differences in IL-10 expression between the treatment groups (Figure 4(d)). There was a significant increase in serum TNF*α* in the MNX-MSC early passage treatment group, compared to the sham-SFM controls and MNX-MSC late passage treatment group (Figure 4(e)). There were no significant differences in serum *β*NGF expression between the groups (Figure 4(f)).

### 3.5. MRI Tracking

**Figure 5:**
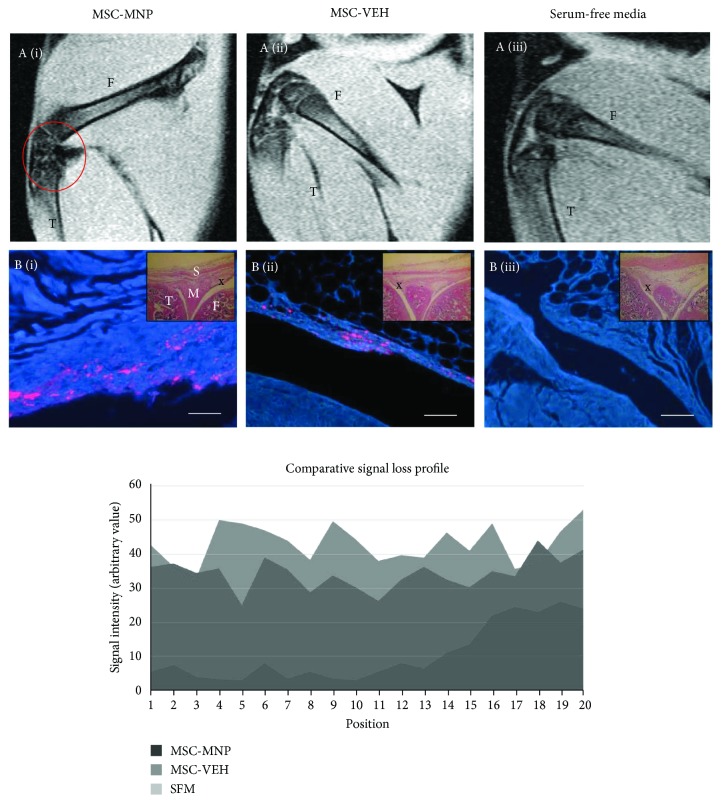
(a): Sagittal MRI scans; Location of SiMAG-labelled cells are depicted as areas of hypointense signal loss and highlighted by the red ring over the synovial cavity. (b) Corresponding histological sections. Fluorescent images correspond to location marked X on H&E images (inset). Implanted DiI-labelled MSCs are shown in red whilst all native materials are show by DAPI in blue. (c) Corresponding MRI signal loss profile. Groups include (i) SiMAG-labelled MSCs (MSC-MNP), (ii) MSCs only (MSC-VEH), and (iii) serum-free media. T: tibia; F: femur; S: synovial lining; M: meniscus. Scale bars = 100 *μ*m.

A subset of rats received intra-articular injection of SiMAG-labelled MSCs (MSC-MNP) for terminal MRI imaging at 29 days post cell implantation. MRI revealed regions of increased hypointensity (black areas) localised to the synovial cavity of rats treated with MSC-MNP (Figure 5). In contrast, no hypointense regions were evident in the groups treated with MSCs (MSC-VEH) or serum-free media (SFM). To further validate these data, signal loss profiles were plotted for the different treatment groups and revealed a significant signal loss in the MSC-MNP group, compared to unlabelled MSCs (MSC-VEH) and SFM which had a relatively high signal intensity across the joint (Figure 5). In a subset of rats, it was confirmed that there were no differences between the effects of SiMAG-labelled MSCs and unlabelled MSCs on pain behaviour (data not shown). H&E staining was used to identify key structural features of the knee joint whilst identifying the location of the fluorescently labelled MSCs. CM-DiI-labelled MSCs were identified in the synovium of all MSC-treated groups (MSC-MNP and MSC-VEH) but not in the SFM-treated group (Figure 5).

## 4. Discussion

This study presents new evidence that passage number of MSCs markedly influences the effects of these cells on pain behaviour following their injection into the knee joint in a surgical model of OA pain. We report that late passage MSCs significantly reduced weight-bearing difference, a surrogate index of pain on loading, whereas early passage MSCs exacerbated weight-bearing difference for a period of 7 days postinjection in the MNX model. Despite the beneficial effects of the late passage MSCs on pain behaviour in the MNX model, there was no evidence for an alteration in the progression of joint pathology or inflammation at the end of the study. Nevertheless, a peripheral site of action of the MSCs was supported by the demonstration that SiMAG-labelled MSCs were detected within the synovial cavity at 29 days postinjection.

As joint degeneration progresses, a variety of surgical procedures can rebuild the degenerated cartilage lesions, but do not necessarily reduce the generalised joint inflammatory processes. Chondrocytes as a cell-based therapy (autologous chondrocyte implantation (ACI)) were successfully developed and used widely over the past 10 years, but this treatment relies on damaging healthy cartilage to provide the cell sources. The therapeutic potential of alternate sources of cells, such as MSCs derived from the bone marrow, which have anti-inflammatory and immunosuppressive properties, has been investigated. Our finding that late passage MSCs attenuated established weight-bearing asymmetry in the MNX model is consistent with the report that intra-articular injection of MSCs reversed pain behaviour compared to pretreatment values, but did not alter structural damage or synovial inflammation in the chemical monosodium iodoacetate model of OA pain [27]. The clinical validity of animal models of OA continues to be debated, both in terms of the aetiology of the joint damage and the temporal progression of the structural changes seen in these models, compared to disease progression in patients. The MNX model of OA is believed to replicate some of the key biomechanical events that lead to clinical joint pathology, as well as displaying many of the features associated with joint pathology in OA (see refs in [28]). Our evidence that intra-articular injection of early passage MSCs exacerbated pain behaviour in the model of OA provides important new knowledge of the conditions under which the therapeutic potential of MSCs for OA pain can be harnessed.

Our data were built upon the previous studies that focused on the potential for MSCs to mediate joint repair. Indeed, therapeutic benefit of intra-articular injection of autologous MSCs has been reported in a surgical model of OA in the goat [17], and more recent studies report beneficial effects of MSCs from bone marrow and adipose in models of OA and extend the initial evidence by demonstrating reparative effects of the cells on the cartilage [29, 30]. The progression of MNX-induced joint pathology was not halted by intra-articular injection of late passage MSCs, suggesting that at least in this model the effects of MSC treatment on pain behaviour are not associated with increased joint repair. Despite this lack of effect on joint pathology, our study did provide evidence for changes in systemic inflammation. There was a trend towards an increase in serum TNF*α* in the MNX model of OA pain, compared to the sham control group at day 42 postsurgery. Interestingly, MNX rats treated with the early passage MSCs had significantly increased serum TNF*α*, compared to the sham group, consistent with the exacerbation of pain behaviour by this treatment. By contrast, serum TNF*α* was significantly lower in MNX rats treated with late passage MSCs, compared to early passage MSCs. These data are consistent with the ability of late passage, but not early passage, MSCs to reduce pain behaviour. Pharmacological studies using comparable methods demonstrated a significant increase in plasma TNF*α* in the MIA model of OA pain compared to control rats, with these changes reversed by an analgesic treatment [31]. Although both IL-10 and NGF were detected in the serum, there were no significant differences between the treatment groups, suggesting that changes in TNF*α* do not reflect a generalised change in inflammation.

Understanding the differences in properties of the early versus late passage MSCs that lead to the behavioural outcomes will help refine MSC treatment strategies. Prolonged *in vitro* culture of bone marrow-derived MSCs leads to a loss of MSC phenotype, multipotency, decreased wound homing properties [32], and self-renewal by around passage 15–20, associated with the onset of cellular senescence [33–36]. As a result, the use of early passage cells is recommended; however, early passage cell populations have increased likelihood of heterogeneity whilst late passage cells retain characteristic markers for MSC phenotype in a selectively more homogenous population [36, 37]. The immunomodulatory properties of MSCs in a long-term culture have been reported. Late passage MSCs of umbilical origin (passage 15) show significant upregulation of anti-inflammatory mediator HMOX-1, a modulator of IL-10 and NO activity [38, 39]; downregulation of proinflammatory IL-1*α*, IL-1*β*, and IFN-*γ*; reduced proliferation of PHA-stimulated peripheral blood mononuclear cells, implicated in expression of proinflammatory cytokines; and no change in expression of TGF-*β*, a cytokine critical to the immunosuppressive capabilities of transfused MSC [39]. It may therefore follow in our study that IFN-*γ* production is impaired and macrophage balance shifts from M1 to M2, as seen previously with *in vivo* MSC transplantation [40]. Production of IL-6 is increased in the late passage human bone marrow-derived MSC compared to early passage cells, whilst CXCL8 levels fall [41]. IL-6 has both anti- and proinflammatory activities and may function here to activate IL-10 and bind to toll-like receptors to inhibit proinflammatory cytokine production, for example, TNF-*α* and IL-1, whilst also providing protection against bacterial proliferation [42, 43]. More analyses of the cytokine profile are needed to define exact pathways in the variation in potential immunomodulation between early and late passage MSCs in our model of OA pain.

There is evidence of other phenotypic shifts in MSCs with prolonged culture which may provide insight. For example, prolonged culture of MSCs reduces STRO-1 and BMP7 expression, changes alkaline phosphatase activity, and reduces osteogenic capability [36, 44, 45]. Earlier studies, examining passage-dependent differences to chondrogenic potential of MSCs, have produced varied results, with some studies reporting a maintenance of the chondrogenic potential of the cells up to passage 20 [46], or increase in COL2A1 and AGC1 expression from P4 to P9 in human adipose-derived adult stem [18]. Another study reported a reduction of chondrogenic capabilities of MSCs at late passage [33, 35, 47]. With an increasing passage number, MSC isolated from synovium shows migratory behaviour similar to chondrocytes, whilst at low passage (*p* < 4), a reduced ability to undergo chondrogenesis was observed [48]. Our results revealed that late passage MSCs attenuated established pain behaviour (weight-bearing asymmetry) in MNX rats, but did not alter MNX-induced joint pathology. This suggests that higher passage cells may have an increased potential for therapeutic application. What is clear is that selection of populations of cells for therapy will depend on the level of preculture with significant changes in phenotype and potential therapeutic activity as a result of prolonged culture. The trade-off between efficacy and cell number generated by scale-up of cell numbers will be an important consideration in a therapeutic design. Future studies of earlier experimental timepoints will shed light on the effects of treatments on the alleviation of pain and moderation of inflammatory response following MSC treatment.

The key to enabling the translation of MSC treatment for OA to the clinic is the tracking of the cells following injection into the knee joint to initially demonstrate effective delivery of cells and to monitor cell retention and biodistribution thereafter. Previously, we have shown that SiMAG, a commercially available superparamagnetic iron oxide nanoparticles (SPIONs), can be used to track the migration of MSCs over 7 days and localise them to the knee joint in a mouse model of rheumatoid arthritis (RA) [20]. Importantly, the same study demonstrated no adverse effects in terms of *in vitro* MSC properties, cell viability, and proliferation as a result of SiMAG labelling. Furthermore, the delivery of SiMAG-labelled MSCs was also well tolerated by mice thereby encouraging the use of SiMAG as an imaging and tracking agent in this current study. Although a number of noninvasive imaging modalities can be applied to track cells post implantation, SPION- and MRI-based tracking techniques benefit from relatively long-term and longitudinal monitoring of implanted cell populations. In a recent study by van Buul et al. [27], implanted MSC populations were monitored by bioluminescence and MRI with MSCs transfected with firefly luciferase and labelled with a particular SPION known as Endorem. A gradual and complete loss in bioluminescence signal was observed over a 3-week period. By contrast, the SPION-based component of this system has the potential to allow SPION-labelled cells to be monitored for up to 12 weeks as demonstrated by 2 independent studies by Jing et al. [49] and Chen et al. [50]. In these studies, Endorem-labelled MSCs and chondrocytes were monitored *in vivo* for up to 12 weeks within the knee joint [49, 50]. Nevertheless, unlike bioluminescence, the viability of implanted cells cannot be determined by MRI-based modalities. In our study, SiMAG-labelled cell was successfully observed up to 29 days post implantation.

## 5. Conclusions

We have demonstrated differences in pain responses in a rat surgical model of OA following cell therapy using populations of MSCs at early and late passages following isolation from the bone marrow. Late passage MSCs significantly reduced weight-bearing difference, a surrogate index of pain on loading (days 31, 35, and 38), whereas early passage MSCs exacerbated weight-bearing difference for a period of 7 days postinjection in the MNX model. Neither treatment altered progression of joint pathology nor inflammation quantified at the end of the study. Our data provide further evidence for the need for characterisation of this cell type prior to clinical use due to its multifunctional nature and the changing phenotype from repair to immunomodulation with time in culture.

## Supplementary Materials

Supplementary materials Supplementary Figure 1: Cell marker profile for early and late passage MSC, characterised through CD105^+^, Sca-1^+^, CD31^−^, CD11b^−^, CD45^−^, CD44^+^, CD34^−^. CD105 is known to vary in expression in murine MSC. In this study, cells at late passage expressed lower levels of CD105 whilst all other markers were consistently expressed. Supplementary Figure 2: Tri-lineage differentiation for early (A) and late (B) passage cells along adipogenic (1), osteogenic (2) and chondrogenic (3) lineages. Positive differentiation was detected for both early and late passage MSC. Scale bars = 200 *μ*m. Supplementary Figure 3: Measurement of cytokines conditioned media from early and late passage cultured MSCs. There were no differences in IL10 levels between the two conditions. TNF*α* was not detected in conditioned medium. Expression of *β*NGF was significantly higher in late passage MSC compared to early passage MSC (Student's *t*-test, *p* < 0.05) (*n* = 6 for all samples). Supplementary Figure 4: In vivo dose response: Signal loss profiles and corresponding sagittal MR images following the implantation of (A) 1 × 10^6^ and (B) 2 × 10^6^ MSCs labelled with (i) 10 *μ*g/ml, (ii) 5 *μ*g/ml, and (iii) 1 *μ*g/ml SiMAG into knee joints of cadaveric rats and compared to the (iv) untreated control. Location of SiMAG labelled cells is depicted as areas of hypointense signal loss and highlighted by the red ring over the synovial cavity.

## References

[1] Glyn-Jones S., Palmer A. J. R., Agricola R. (2015). Osteoarthritis. *Lancet*.

[2] Neogi T., Zhang Y. (2013). Epidemiology of osteoarthritis. *Rheumatic Diseases Clinics of North America*.

[3] Conaghan P. G., Peloso P. M., Everett S. V. (2015). Inadequate pain relief and large functional loss among patients with knee osteoarthritis: evidence from a prospective multinational longitudinal study of osteoarthritis real-world therapies. *Rheumatology*.

[4] Graven-Nielsen T., Wodehouse T., Langford R. M., Arendt-Nielsen L., Kidd B. L. (2012). Normalization of widespread hyperesthesia and facilitated spatial summation of deep-tissue pain in knee osteoarthritis patients after knee replacement. *Arthritis & Rheumatism*.

[5] Roberts S., Genever P., McCaskie A., De Bari C. (2011). Prospects of stem cell therapy in osteoarthritis. *Regenerative Medicine*.

[6] de Lange-Brokaar B. J., Ioan-Facsinay A., Yusuf E. (2015). Association of pain in knee osteoarthritis with distinct patterns of synovitis. *Arthritis & Rheumatology*.

[7] Bouffi C., Djouad F., Mathieu M., Noël D., Jorgensen C. (2009). Multipotent mesenchymal stromal cells and rheumatoid arthritis: risk or benefit?. *Rheumatology*.

[8] Kastrinaki M. C., Sidiropoulos P., Roche S. (2008). Functional, molecular and proteomic characterisation of bone marrow mesenchymal stem cells in rheumatoid arthritis. *Annals of the Rheumatic Diseases*.

[9] Ringe J., Sittinger M. (2009). Tissue engineering in the rheumatic diseases. *Arthritis Research & Therapy*.

[10] Henning T. D., Gawande R., Khurana A. (2012). Magnetic resonance imaging of ferumoxide-labeled mesenchymal stem cells in cartilage defects: in vitro and in vivo investigations. *Molecular Imaging*.

[11] da Silva Meirelles L., Fontes A. M., Covas D. T., Caplan A. I. (2009). Mechanisms involved in the therapeutic properties of mesenchymal stem cells. *Cytokine & Growth Factor Reviews*.

[12] Malfait A. M., Little C. B., McDougall J. J. (2013). A commentary on modelling osteoarthritis pain in small animals. *Osteoarthritis and Cartilage*.

[13] Mapp P. I., Sagar D. R., Ashraf S. (2013). Differences in structural and pain phenotypes in the sodium monoiodoacetate and meniscal transection models of osteoarthritis. *Osteoarthritis and Cartilage*.

[14] Vincent T. L., Williams R. O., Maciewicz R., Silman A., Garside P. (2012). Mapping pathogenesis of arthritis through small animal models. *Rheumatology*.

[15] Suhaeb A. M., Naveen S., Mansor A., Kamarul T. (2012). Hyaluronic acid with or without bone marrow derived-mesenchymal stem cells improves osteoarthritic knee changes in rat model: a preliminary report. *Indian Journal of Experimental Biology*.

[16] Kim J. E., Lee S. M., Kim S. H. (2014). Effect of self-assembled peptide–mesenchymal stem cell complex on the progression of osteoarthritis in a rat model. *International Journal of Nanomedicine*.

[17] Murphy J. M., Fink D. J., Hunziker E. B., Barry F. P. (2003). Stem cell therapy in a caprine model of osteoarthritis. *Arthritis & Rheumatism*.

[18] Estes B. T., Wu A. W., Storms R. W., Guilak F. (2006). Extended passaging, but not aldehyde dehydrogenase activity, increases the chondrogenic potential of human adipose-derived adult stem cells. *Journal of Cellular Physiology*.

[19] Peister A., Mellad J. A., Larson B. L., Hall B. M., Gibson L. F., Prockop D. J. (2004). Adult stem cells from bone marrow (MSCs) isolated from different strains of inbred mice vary in surface epitopes, rates of proliferation, and differentiation potential. *Blood*.

[20] Markides H., Kehoe O., Morris R. H., El Haj A. J. (2013). Whole body tracking of superparamagnetic iron oxide nanoparticle-labelled cells–a rheumatoid arthritis mouse model. *Stem Cell Research & Therapy*.

[21] Gnecchi M., Melo L. G. (2009). Bone marrow-derived mesenchymal stem cells: isolation, expansion, characterization, viral transduction, and production of conditioned medium. *Methods in Molecular Biology*.

[22] Ashraf S., Mapp P. I., Burston J., Bennett A. J., Chapman V., Walsh D. A. (2014). Augmented pain behavioural responses to intra-articular injection of nerve growth factor in two animal models of osteoarthritis. *Annals of the Rheumatic Diseases*.

[23] Christiansen C. L., Stevens-Lapsley J. E. (2010). Weight-bearing asymmetry in relation to measures of impairment and functional mobility for people with knee osteoarthritis. *Archives of Physical Medicine and Rehabilitation*.

[24] Sagar D. R., Ashraf S., Xu L. (2014). Osteoprotegerin reduces the development of pain behaviour and joint pathology in a model of osteoarthritis. *Annals of the Rheumatic Diseases*.

[25] Gerwin N., Bendele A. M., Glasson S., Carlson C. S. (2010). The OARSI histopathology initiative–recommendations for histological assessments of osteoarthritis in the rat. *Osteoarthritis and Cartilage*.

[26] Mankin H. J., Dorfman H., Lippiello L., Zarins A. (1971). Biochemical and metabolic abnormalities in articular cartilage from osteo-arthritic human hips: II. Correlation of morphology with biochemical and metabolic data. *The Journal of Bone & Joint Surgery*.

[27] van Buul G. M., Siebelt M., Leijs M. J. C. (2014). Mesenchymal stem cells reduce pain but not degenerative changes in a mono-iodoacetate rat model of osteoarthritis. *Journal of Orthopaedic Research*.

[28] Ashraf S., Mapp P. I., Walsh D. A. (2011). Contributions of angiogenesis to inflammation, joint damage, and pain in a rat model of osteoarthritis. *Arthritis and Rheumatism*.

[29] Mokbel A. N., el Tookhy O. S., Shamaa A. A., Rashed L. A., Sabry D., el Sayed A. M. (2011). Homing and reparative effect of intra-articular injection of autologus mesenchymal stem cells in osteoarthritic animal model. *BMC Musculoskeletal Disorders*.

[30] ter Huurne M., Schelbergen R., Blattes R. (2012). Antiinflammatory and chondroprotective effects of intraarticular injection of adipose-derived stem cells in experimental osteoarthritis. *Arthritis & Rheumatism*.

[31] Burston J. J., Sagar D. R., Shao P. (2013). Cannabinoid CB_2_ receptors regulate central sensitization and pain responses associated with osteoarthritis of the knee joint. *PLoS One*.

[32] Rombouts W. J. C., Ploemacher R. E. (2003). Primary murine MSC show highly efficient homing to the bone marrow but lose homing ability following culture. *Leukemia*.

[33] Banfi A., Muraglia A., Dozin B., Mastrogiacomo M., Cancedda R., Quarto R. (2000). Proliferation kinetics and differentiation potential of ex vivo expanded human bone marrow stromal cells: implications for their use in cell therapy. *Experimental Hematology*.

[34] Somasundaram I., Mishra R., Radhakrishnan H., Sankaran R., Garikipati V. N. S., Marappagounder D. (2015). Human adult stem cells maintain a constant phenotype profile irrespective of their origin, basal media, and long term cultures. *Stem Cells International*.

[35] Bonab M., Alimoghaddam K., Talebian F., Ghaffari S., Ghavamzadeh A., Nikbin B. (2006). Aging of mesenchymal stem cell in vitro. *BMC Cell Biology*.

[36] Tondreau T., Lagneaux L., Dejeneffe M. (2004). Isolation of BM mesenchymal stem cells by plastic adhesion or negative selection: phenotype, proliferation kinetics and differentiation potential. *Cytotherapy*.

[37] Tropel P., Noël D., Platet N., Legrand P., Benabid A. L., Berger F. (2004). Isolation and characterisation of mesenchymal stem cells from adult mouse bone marrow. *Experimental Cell Research*.

[38] Bach F. H. (2005). Heme oxygenase-1: a therapeutic amplification funnel. *FASEB Journal*.

[39] Zhuang Y., Li D., Fu J., Shi Q., Lu Y., Ju X. (2015). Comparison of biological properties of umbilical cord‑derived mesenchymal stem cells from early and late passages: immunomodulatory ability is enhanced in aged cells. *Molecular Medicine Reports*.

[40] Hajkova M., Javorkova E., Zajicova A., Trosan P., Holan V., Krulova M. (2017). A local application of mesenchymal stem cells and cyclosporine A attenuates immune response by a switch in macrophage phenotype. *Journal of Tissue Engineering and Regenerative Medicine*.

[41] Briquet A., Dubois S., Bekaert S., Dolhet M., Beguin Y., Gothot A. (2010). Prolonged ex vivo culture of human bone marrow mesenchymal stem cells influences their supportive activity toward NOD/SCID-repopulating cells and committed progenitor cells of B lymphoid and myeloid lineages. *Haematologica*.

[42] Nishimoto N. (2006). Interleukin-6 in rheumatoid arthritis. *Current Opinion in Rheumatology*.

[43] van der Poll T., Keogh C. V., Guirao X., Buurman W. A., Kopf M., Lowry S. F. (1997). Interleukin-6 gene-deficient mice show impaired defense against pneumococcal pneumonia. *The Journal of Infectious Diseases*.

[44] Chen J., Sotome S., Wang J., Orii H., Uemura T., Shinomiya K. (2005). Correlation of in vivo bone formation capability and in vitro differentiation of human bone marrow stromal cells. *Journal of Medical and Dental Sciences*.

[45] Vacanti V., Kong E., Suzuki G., Sato K., Canty J. M., Lee T. (2005). Phenotypic changes of adult porcine mesenchymal stem cells induced by prolonged passaging in culture. *Journal of Cellular Physiology*.

[46] Yoo J. U., Barthel T. S., Nishimura K. (1998). The chondrogenic potential of human bone-marrow-derived mesenchymal progenitor cells. *Journal of Bone & Joint Surgery*.

[47] Li Z., Liu C., Xie Z. (2011). Epigenetic dysregulation in mesenchymal stem cell aging and spontaneous differentiation. *PLoS One*.

[48] Tan A. R., Alegre-Aguarón E., O'Connell G. D. (2015). Passage-dependent relationship between mesenchymal stem cell mobilization and chondrogenic potential. *Osteoarthritis and Cartilage*.

[49] Jing X.-H., Yang L., Duan X. J. (2008). In vivo MR imaging tracking of magnetic iron oxide nanoparticle labeled, engineered, autologous bone marrow mesenchymal stem cells following intra-articular injection. *Joint, Bone, Spine*.

[50] Chen J., Wang F., Zhang Y. (2012). In vivo tracking of superparamagnetic iron oxide nanoparticle labeled chondrocytes in large animal model. *Annals of Biomedical Engineering*.

