# Supplementary material for: Therapeutic Benefit for Late, but Not Early, Passage Mesenchymal Stem Cells on Pain Behaviour in an Animal Model of Osteoarthritis
Source: Stem Cells Int. 2017 Dec 24;2017:2905104. doi: 10.1155/2017/2905104 (PMC5757143; doi:10.1155/2017/2905104)
Supplement: Supplementary materials — Supplementary Figure 1: Cell marker profile for early and late passage MSC, characterised through CD105+, Sca-1+, CD31−, CD11b−, CD45−, CD44+, CD34−. CD105 is known to vary in expression in murine MSC. In this study, cells at late passage expressed lower levels of CD105 whilst all other markers were consistently expressed. Supplementary Figure 2: Tri-lineage differentiation for early (A) and late (B) passage cells along adipogenic (1), osteogenic (2) and chondrogenic (3) lineages. Positive differentiation was detected for both early and late passage MSC. Scale bars = 200 μm. Supplementary Figure 3: Measurement of cytokines conditioned media from early and late passage cultured MSCs. There were no differences in IL10 levels between the two conditions. TNFα was not detected in conditioned medium. Expression of βNGF was significantly higher in late passage MSC compared to early passage MSC (Student's t-test, p < 0.05) (n = 6 for all samples). Supplementary Figure 4: In vivo dose response: Signal loss profiles and corresponding sagittal MR images following the implantation of (A) 1 × 10^6^ and (B) 2 × 10^6^ MSCs labelled with (i) 10 μg/ml, (ii) 5 μg/ml, and (iii) 1 μg/ml SiMAG into knee joints of cadaveric rats and compared to the (iv) untreated control. Location of SiMAG labelled cells is depicted as areas of hypointense signal loss and highlighted by the red ring over the synovial cavity. [file 2905104.f1.pdf]

Supplementary Methods:

Cell Isolation and Expansion

Early passage murine MSCs were isolated from Balb/c mice and late passage cells were isolated from C57Bl/6 mice as previously described [19]. Briefly, bone marrow cells were collected by flushing femurs and tibiae, and cells were plated out in cell isolation media (CIM) (RPMI-1640) (Gibco,UK) with 9% fetal bovine serum (Gibco,UK), 9% horse serum (Gibco,UK) and 1% penicillin-streptomycin at 37^oC in 5% CO_2. After 24 hours, nonadherent cells were removed and 4 weeks later, cells were re-plated at 100 cells per cm^2 in complete expansion media (CEM) (Iscove Modified Dulbecco Medium (IMDM)) (Gibco, UK) supplemented with 9% fetal bovine serum (Gibco, UK), 9% horse serum (Gibco, UK) and 1% penicillin-streptomycin for MSCs expansion.

The number of cells injected was based on previously published studies [16, 26], showing that $1x10^6$ cells had beneficial effects in rodent models of OA. In line with MRI visibility thresholds, we slightly increased the number of cells to $1.5x10^6$.

Cell Characterisation

$1x10^5$ MSCs were analysed for membrane receptor expression. Antibodies used in this study were as follows: anti-mouse CD31 (PECAM-1) PE, anti-human/mouse CD44 PE, anti-mouse CD11b PE, anti-mouse CD45 PE, anti-mouse CD105 PE, anti-mouse Ly-6A(Sca-1)PE (all from eBioscience). A minimum of 10,000 events were recorded for each analysis, using FACScan flow cytometer and analysed using CellQuestPro software (Becton Dickinson, Oxford, UK). Cells were examined for their ability to form colonies and to differentiate into osteocytes, chondrocytes and adipocytes, as described [19]. Only MSCs showing full differentiation potential were used for the experiments (Supplementary Figure 1, Supplementary Figure 2). The CFU-F assay was performed as previously described [19].

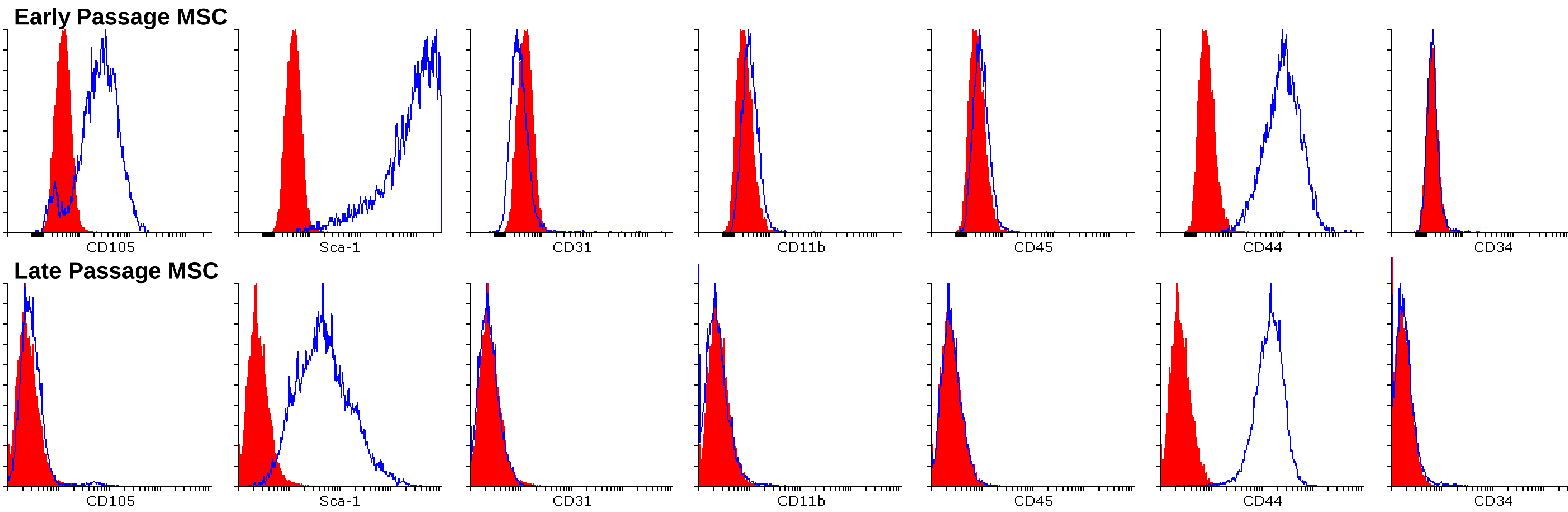

Supplementary Figure 1:

Cell marker profile for early and late passage MSC, characterised through $CD105^{+}$, $Sca\text{-}1^{+}$, $CD31^{-}$, $CD11b^{-}$, $CD45^{-}$, $CD44^{+}$, $CD34^{-}$. CD105 is known to vary in expression in murine MSC. In this study, cells at late passage expressed lower levels of CD105 whilst all other markers were consistently expressed.

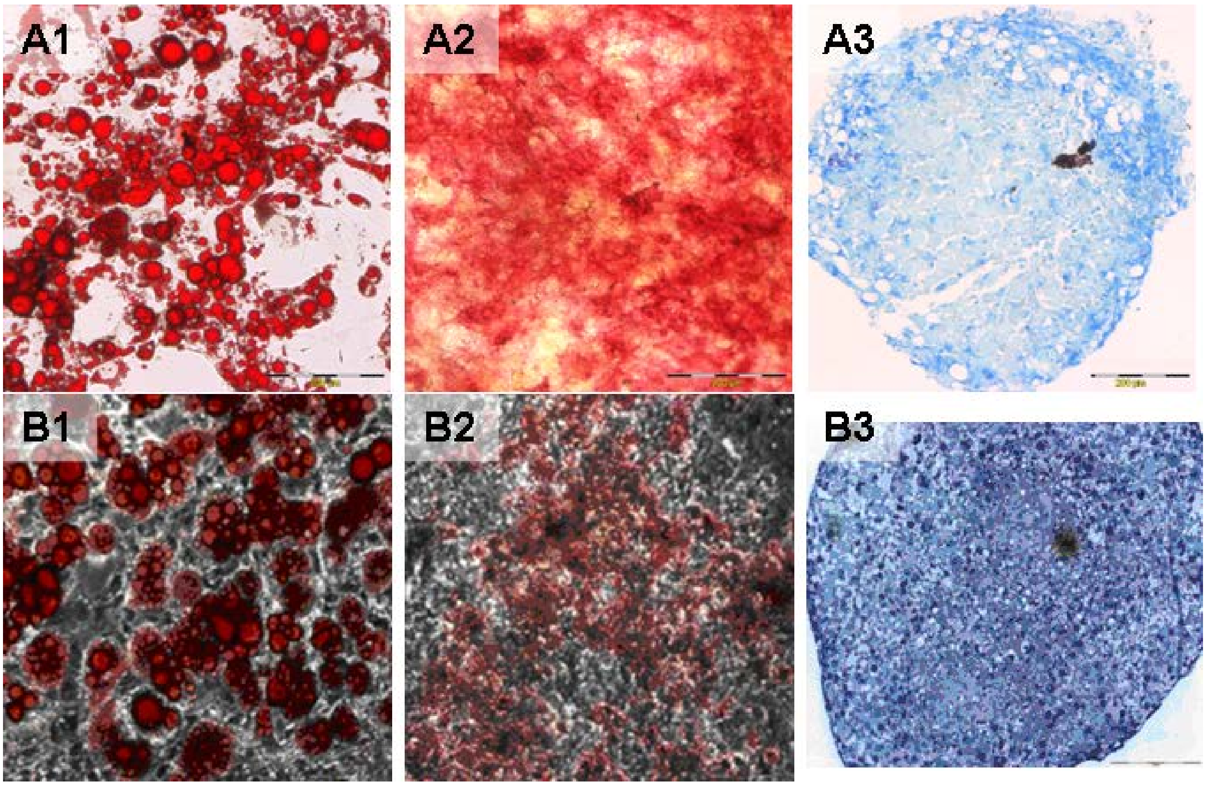

Supplementary Figure 2:

Tri-lineage differentiation for early (A) and late (B) passage cells along adipogenic (1), osteogenic (2) and chondrogenic (3) lineages. Positive differentiation was detected for both early and late passage MSC. Scale bars = 200µm.

Pain Behaviour Assessment

Weight-distribution through the left (ipsilateral) and right (contralateral) knees were assessed using an incapacitance tester (Linton Instrumentation, U.K.). Hind-paws were placed on separate sensors and the force (in grams) exerted by each hind limb was recorded and averaged over a period of 3 seconds as previously described [23]. Each data point is the mean of three separate 3 sec measurements. Naïve rats distribute their weight evenly between both paws, following joint injury changes in weight distribution can be used an indicator of joint discomfort and associated pain in the injured knee. Changes in hindpaw withdrawal thresholds (PWTs) were assessed using von Frey monofilaments (Semmes-Weinstein monofilaments of bending forces 1, 1.4, 2, 4, 6, 8, 10 and 15 g). Rats were placed in transparent plastic cubicles on a mesh floored table. Following a period of acclimatization calibrated Von Frey monofilaments were applied, in ascending order of bending force, to the plantar surface of both hind-paws. Each von Frey was applied for a 3 sec period. Once a withdrawal reflex was established, the paw was re-tested with the next descending von Frey

monofilament until no response occurred, known as the “up down method” [13]. The lowest weight of monofilament which elicited a withdrawal reflex was noted as the PWT.

MRI data analysis

Bruker MRI files were converted to Analyze files using Bru2Anz converter and subsequently processed using MRIcron. Sagittal knee slices were assessed to identify 3 slices wheres all the anatomical structures of the knee were in full form and the signal intensity (SI) at 20 points of equal intervals across each knee measured. The average SI of the 3 slices were then averaged, plotted and compared.

Histology for study 1

Further histology was performed to validate the MRI results from study 1. Here, joints were fixed in neutral buffered formal saline, and decalcified with formic acid at 4 ^{o}C before embedding in paraffin. Mid-sagittal serial sections (4 µm thickness) were obtained using a Buehler Isomet low speed saw with a diamond tipped blade. Joint sections were prepared for subsequent staining by initially de-waxing sections in 100 % Xylene for 5 mins. Samples were then rehydrated in 100 % ethanol for a further 2 min followed by washes in PBS for 5 min prior to subsequent staining with H&E and the fluorescent dye DAPI (1:200 dilution prepared in PBS).

Knee Joint histopathology

Articular cartilage surface integrity was scored using the OARSI cartilage histopathology assessment system, modified to score 3 coronal sections at 200 um intervals from the anterior half of the knee, corresponding to the region evaluated for osteoclast numbers [24]. Cartilage histopathology and a total joint damage score was calculated as previously described [23]. Osteophytosis was scored on a scale from 0-3, where 0 (no osteophyte), 1 (<40µm), 2 (40-160µm), 3 (osteophyte >160µm). As previously described [23], synovial inflammation was scored according to the thickness of the synovial lining layer and synovial cellularity in the medial and lateral tibiofemoral compartment: 0 Lining cell layer 1-2 cells thick; 1 Lining cell layer 3-5 cells thick; 2 Lining cell layer 6-8 cells thick and/or mild increase in cellularity; 3 Lining cell layer > 9 cells thick and/or severe increase in cellularity.

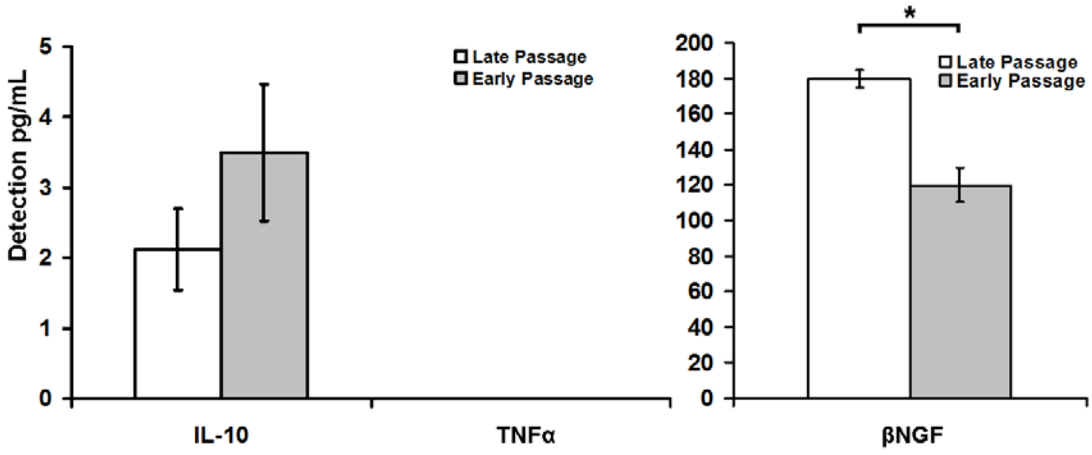

Supplementary Figure 3:

Measurement of cytokines conditioned media from early and late passage cultured MSCs. There were no differences in IL10 levels between the two conditions. TNFα was not detected in conditioned medium. Expression of βNGF was significantly higher in late passage MSC compared to early passage MSC (student's t-test, $p<0.05$) (n=6 for all samples).

MRI in vivo dose response

The optimal cell number and SiMAG ratio for long term MRI tracking was determined by injecting 1×10^6 or 2×10^6 MSCs labeled with 0, 1, 5 and 10 µg/ml SiMAG into the joint of non-arthritic cadaveric rats and MR imaging (Supplementary Figure 4). Hypointense regions of signal loss (corresponding to a relatively low signal intensity value) were observed over the synovial joint when either $1x10^6$ MSCs labelled with 5 and 10 µg/ml of SiMAG and $2x10^6$ MSCs labelled with 1, 5 and 10 µgFe/ml of SiMAG. On this basis 1.5×10^6 cells labelled with 10 µg/ml SiMAG was considered optimal for the in vivo MRI tracking studies to ensure good MRI visibility over a prolonged period of time.

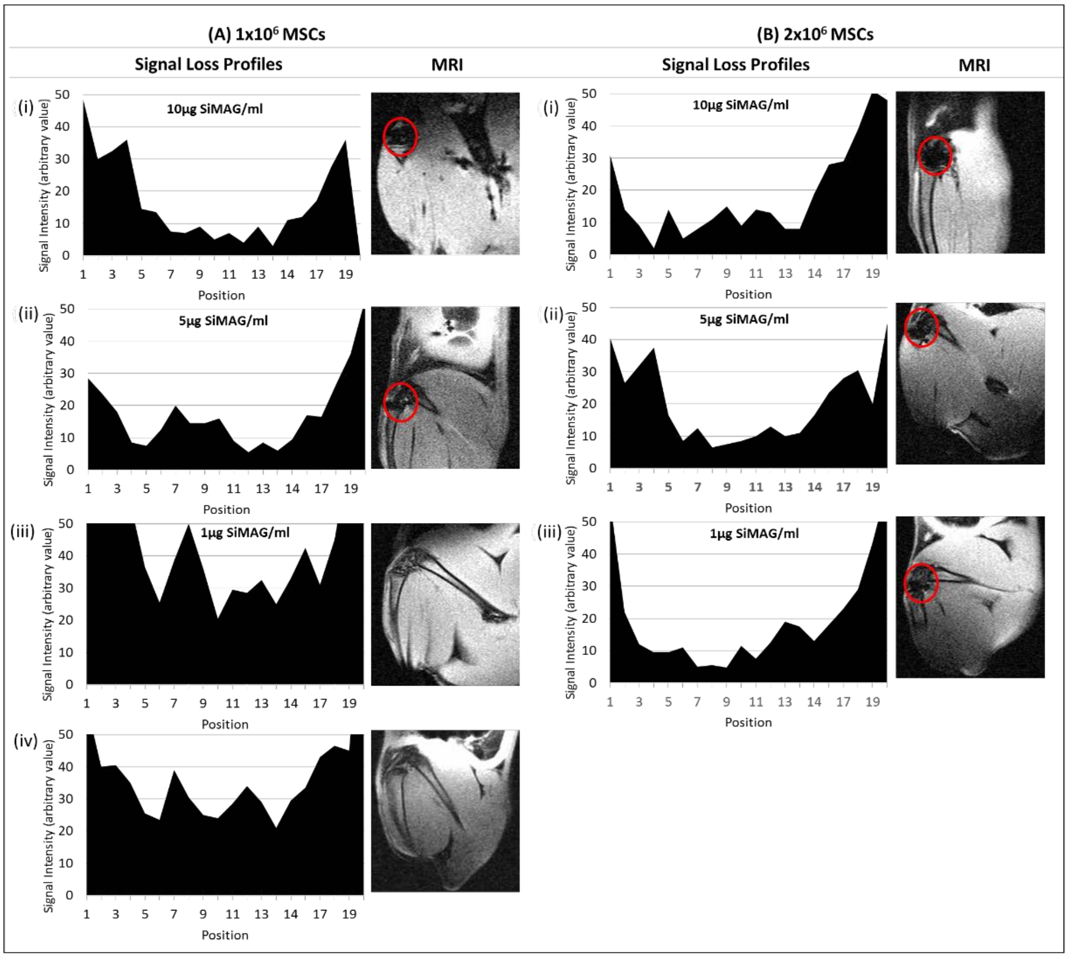

Supplementary Figure 4: In vivo dose response:

Signal loss profiles and corresponding sagittal MR images following the implantation of **(A)** $1x10^6$ and **(B)** $2x10^6$ MSCs labelled with: **(i)** 10 µg/ml, **(ii)** 5 µg/ml and **(iii)** 1 µg/ml SiMAG into knee joints of cadaveric rats and compared to the **(iv)** untreated control. Location of SiMAG labelled cells are depicted as areas of hypointense signal loss and highlighted by the red ring over the synovial cavity.
